# Sunitinib and other targeted therapies for renal cell carcinoma

**DOI:** 10.1038/sj.bjc.6606061

**Published:** 2011-01-25

**Authors:** T Powles, S Chowdhury, R Jones, M Mantle, P Nathan, A Bex, L Lim, T Hutson

**Affiliations:** 1 Department of Medical Oncology, St Bartholomew's Hospital London, Barts and the London NHS trust, Charterhouse Square, London, UK; 2 Department of Medical Oncology, The Beatson Hospital Glasgow, UK; 3 Department of Medical Oncology, Guy's and St Thomas’ Hospital, London, UK; 4 Department of Medical Oncology, Baylor University Medical Centre, Dallas, TX, USA; 5 Department of Medical Oncology, Mount Vernon Hospital, London, UK; 6 Department of Medical Oncology, National Cancer Institute, Amsterdam, The Netherlands

**Keywords:** renal cancer, sunitinib, mTOR, VEGF

## Abstract

Targeted therapy has radically altered the way metastatic renal cancer is treated. Six drugs are now licensed in this setting, with several other agents under evaluation. Sunitinib is currently the most widely used in the first line setting with impressive efficacy and an established toxicity profile. However, as further randomised studies report and as newer drugs become available this may change. In this review, we address our current understanding of targeted therapy in renal cancer. We also discuss areas in which our knowledge is incomplete, including the identification of correlative biomarkers and mechanisms of drug resistance. Finally, we will describe the major areas of clinical research that will report over the next few years.

The treatment of metastatic clear cell renal carcinoma (RCC) has dramatically changed over the last 5 years. This has been driven by two groups of targeted agents; namely vascular endothelial growth factor (VEGF)-targeted therapies and mammalian target of rapamycin (mTOR) inhibitors. Both, first and second line treatment is of proven benefit and these agents have replaced immune therapies that were previously standard of care for metastatic RCC (Motzer and Bukowski, 2006; Motzer *et al*, 2009).

Sunitinib is a multitargeted tyrosine kinase inhibitor (TKI) that predominantly targets VEGF (Figure 1). It also has ‘off target’ effects, involving other tyrosine kinases that may account for some of its activity and toxicity (Motzer and Bukowski, 2006). The pivotal trial of sunitinib was published in 2006; and subsequently it has established itself as standard first line therapy (Motzer *et al*, 2009). The exact mechanism of its activity in RCC remains unknown, and it is not yet possible to identify specific cohorts of patients who benefit from therapy. Additionally, sunitinib and the other drugs are only effective in controlling the disease for a limited period before progression occurs. Therefore, an important area of research is investigating mechanisms of resistance.

Sunitinib is one of a number of VEGF-targeted agents with activity in this setting. Four drugs including sunitinib have been approved by the FDA for the treatment of metastatic RCC. The other three agents are sorafenib, pazopanib and bevacizumab (Escudier *et al*, 2007a, 2007b; Rini *et al*, 2008a, 2008b; Sternberg *et al*, 2009). Newer agents under investigation in ongoing randomised trials include tivozinib, dovitinib and axtitinib. It is hoped that these agents will show improved activity, decreased toxicity or set a benchmark as second or third line therapy. However, none of these trials are aimed at specific subsets of patients with metastatic clear cell RCC.

The other major group of targeted agents in renal cancer is the mTOR inhibitors. These include temsirolimus and everolimus that are both widely used and have proven activity (Hudes *et al*, 2007; Motzer *et al*, 2008a, 2008b). Once again there has been a failure to identify specific groups of patients that benefit from these agents, and although these agents offer initial benefit, resistance occurs.

This review addresses our current understanding of the role of targeted therapy in renal cancer. It will also highlight areas in which our understanding is incomplete and finally will describe the major areas of clinical research, which will report over the next few years.

## Histology and prognosis in metastatic renal cancer

**Figure 1:**
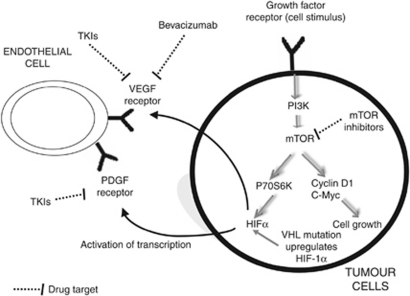
Diagrammatic representation of the mechanism of action of targeted therapy in clear cell renal cancer. This figure shows how HIF-1 is unregulated within the clear cell renal cancer tumour cell by two mechanisms. This upregulation in turn stimulates receptors on endothelial cells (VEGF and PDGF). Dotted lines denote the drug targets. Off-target effects on the stroma and tumour cells are not represented.

Renal cell carcinoma consists of a number of histological subtypes, the most common of which is clear cell RCC. It is characterised by a mutation to the Von Hippel Lindau protein that is linked to both regulation of HIF and VEGF (Na *et al*, 2003) (Figure 1). Papillary RCC accounts for the bulk of the remaining ‘non-clear cell’ tumours. The majority of trials focus on the clear cell population in which HIF/VEGF appears to be the dominant tumour biology (Rini *et al*, 2008a, 2008b).

The most commonly used method for predicting outcome of patients with metastatic renal cancer is the Memorial Sloan-Kettering Prognostic score. It stratifies patients into three groups (good, intermediate and poor) depending on a number of factors, including lactate dehydrogenase, performance status, serum calcium, haemoglobin and time since diagnosis to treatment (Motzer and Bukowski, 2006). Although designed before the introduction of targeted therapy it has been validated in the TKI era (Motzer *et al*, 2008; Heng *et al*, 2009).

## Sunitinib in metastatic renal cancer

Sunitinib is superior to interferon in the first line treatment of clear cell RCC. In the pivotal study the progression-free survival (PFS) was 11 months for sunitinib *vs* 5 months for interferon (hazard ratio (HR): 0.53; (95% CI: 0.45–0.64); *P*<0.001) (Motzer *et al*, 2007, 2009). Most of the study patients were MSKCC good and intermediate risk, had a good performance status and a nephrectomy before treatment. The majority of patients on the interferon arm subsequently received sunitinib (33%) or other VEGF-signalling inhibitors (32%) confounding the overall survival (OS) data (26.4 months for sunitinib *vs* 21.8 months for interferon (HR: 0.821; 95% CI: 0.673–1.001; *P*=0.051). Subsequent analysis of those patients who did not cross over to sunitinib showed an OS of 14 months for interferon and 28 months for sunitinib (Flanigan *et al*, 2001; Motzer *et al*, 2009). The benefits of sunitinib were present in all three MSKCC prognostic groups, although the numbers in the poor-risk category were small (<10% of the study population) resulting in statistically insignificant data. Subsequent data from a large expanded access series reproduced the PFS results (10.9 months; 95% CI: 10.3–11.2 months), with an OS of 18.4 months (95%CI: 17.4–19.2 months) underlining the practise changing nature of these results (Gore *et al*, 2009).

There is a lack of randomised data for sunitinib in the non-clear cell population. The most comprehensive data comes from the expanded access cohort in which the PFS and OS were 7.8 (95% CI: 6.3–8.3) months and 13.4 (95% CI: 10.7–14.9) months, respectively (Gore *et al*, 2009). A further retrospective analysis supported the use of sunitinib in this setting with a PFS of 11.9 months (Choueiri *et al*, 2008).

The most common toxicities encountered with sunitinib included (grade 3 or more) hypertension (12%), fatigue (11%), diarrhoea (9%) and hand–foot syndrome (9%). In the landmark phase III study 50% of the patients required a dose reduction of sunitinib due to these events (Motzer *et al*, 2008a, 2008b). A recent meta-analysis identified a relationship between steady-state area under the curve of sunitinib and time to tumour progression, toxicity and OS (Houk *et al*, 2009) suggesting increased exposure to sunitinib appears to be associated with improved clinical outcomes, as well as increased risk of adverse effects. Therefore, attempts should initially be made to address toxicity before dose reductions are made. Sunitinib is currently given at 50 mg for 4 consecutive weeks followed by a 2-week treatment-free interval. Phase II data investigating continuous dosing at 37.5 mg showed similar toxicity profile to the 50 mg intermittent dose (Escudier *et al*, 2009a, 2009b, 2009c). A randomized phase II study comparing the standard 50 mg regimen with continuous 37.5 mg dosing is exploring this regimen formally and will report in 2010.

## Predicting clinical benefit with targeted therapy

A number of prognostic biomarkers have been identified that correlate with a poor outcome with sunitinib. These include the MSKCC prognostic criteria, raised platelet/neutrophil levels at diagnosis (Heng *et al*, 2009; Motzer *et al*, 2008a, 2008b). However, it has not been possible to identify biomarkers or imaging modalities that predict clinical benefit with specific agents. Sunitinib causes dynamic changes to cytokine expression and growth factors; however, it remains unclear whether these predict clinical benefit (Rini *et al*, 2008a, 2008b). Further work in this area is required.

Although widely used, response rates using CT (RECIST criteria) do not correlate with outcome (Kontovinis *et al*, 2009). Other methods of correlating CT changes with outcome, such as the Choi criteria, which measures tumour attenuation (Hounsfield units), as well as changes in two-dimensional size, are under investigation and hold promise (van der Veldt *et al*, 2010). Other imagining modalities, such as dynamic contrast enhanced MRI and FDG-PET, are also being investigated (Hahn *et al*, 2008).

Perhaps the most promising current biomarker is the presence of treatment-associated hypertension, which in retrospective analysis, appears to correlate with outcome (Rixe *et al*, 2008). This finding is under investigation in prospective studies with axitinib. It raises a number of issues, for example, is this finding a pharmacokinetic or pharmacodynamic affect and if the hypertension is associated with clinical benefit how aggressively should it be managed?

The optimal way of identifying predictive rather than prognostic biomarkers in this setting is with prospective studies comparing VEGF-targeted agents with other classes of drug, such as the mTOR inhibitors. Using this approach it is possible to determine whether subgroups benefit from the specific agents rather than just identifying prognostic markers. Clinical trials such as RECORD 3 that compares sunitinib and everolimus are the closest we have to this design, although the identification of biomarkers in not the primary endpoint and the translational component is, therefore, not comprehensive.

## Other VEGF-targeted agents

(Table 1) Sorafenib and bevacizumab were both developed at approximately the same time as sunitinib in RCC. Sorafenib is associates with a PFS benefit as second line therapy after interferon failure (Escudier *et al*, 2007a, 2007b). However, a first line sorafenib *vs* interferon randomised phase II study showed no benefit for sorafenib, making it less attractive in this setting (Escudier *et al*, 2009a, 2009b, 2009c).

Two randomised phase III studies investigated bevacizumab in combination with interferon *vs* interferon alone as first line treatment for metastatic RCC (Escudier *et al*, 2007a, 2007b; Rini *et al*, 2008a, 2008b). These studies focused on the clear cell population and consisted of predominantly good and intermediate risk patients. Both studies showed a benefit for the combination in terms of PFS (5.1 *vs* 8.5; 5.4 *vs* 10.2), with the OS for in the bevacizumab arm of 18.3 and 22.9 months ref.

More recently Pazopanib has received FDA and EMA approval. In the pivotal randomised phase III study the PFS for the pazopanib was 11.1 months compared with 2.8 months for placebo for previously untreated patients (HR: 0.40; 95% CI: 0.27, 0.60; *P*<0.001) (Sternberg *et al*, 2009). The toxicity profile for pazopanib may be different to the other VEGF TKIs with the most common adverse event being diarrhoea (52% 4% Grade 3 or 4), hypertension (40% 4% Grade 3 or 4), hair colour changes (38%) and nausea (26% <1% Grade 3 or 4). The incidence of fatigue and ‘hand and foot syndrome’ appears to be relatively low (grade 3 or more in 5 and 2%, respectively). However, abnormalities in liver function tests (>3 × normal) occurred in 20% of patients (Sternberg *et al*, 2009; Hutson *et al*, 2010). These factors may help select the optimal agent for individuals. A pivotal study comparing pazopanib and sunitinib in the first line setting has closed recruitment and will report in 2011.

Although direct comparison of the above agents is not currently possible because of the lack of direct randomized studies in the first line setting, indirect comparison is possible but flawed in nature (Table 1). Nevertheless sunitinib has set a benchmark for both overall and PFS here, although the PFS for pazopanib is also 11 month and is, therefore, promising. In view of the fact that sorafenib did not appear superior to interferon it is more difficult to recommend this agent above those with proven superiority to interferon, such as bevacizumab (with interferon) and sunitinib (Escudier *et al*, 2009a, 2009b, 2009c).

## mTOR inhibitors

Everolimus and temsirolimus are both mTOR inhibitors (TORC1) that have been investigated and are widely used in metastatic RCC (Hudes *et al*, 2007; Motzer *et al*, 2009) (Figure 1). Temsirolimus was investigated in untreated poor risk disease. Results of this study showed prolonged OS for temsirolimus compared with interferon (HR for death: 0.73; 95% CI: 0.58–0.92; *P*=0.01). However, the combination of temsirolimus and interferon together did not show significant benefit over temsirolimus alone. It is speculated that this may be because of the dose intensity achieved in this arm of the study. Subset analysis showed significant benefit for non-clear cell population that is of particular interest and warrants further investigation. The drug was relatively well tolerated with fewer serious adverse events in the temsirolimus group than in the interferon group (*P*=0.02).

Everolimus is the only agent with positive randomised data after TKI failure in RCC (Motzer *et al*, 2009). RECORD 1 compared the effects of everolimus and placebo in patients who had previously received at least one line of targeted therapy. The PFS strongly favoured everolimus (HR: 0.30; 95% CI: 0.22–0.40; *P*<0.0001). Perhaps, the most significant toxicity seen was pneumonitis (any grade 8%), which requires particular attention with using this agent.

## VEGF inhibitors as second line therapy in RCC

Although phase III data support the use of sorafenib as second line therapy, this study conducted in the era of immune therapy and (Escudier *et al*, 2007a, 2007b), there is no randomised data to support further VEGF TKI therapy after failure of initial targeted therapy. However, phase II data suggest that there may be non-cross resistance between these agents (Rini *et al*, 2009; Sablin *et al*, 2009). Therefore, the use of sequential VEGF-targeted therapy may be beneficial. A number of randomised phase II and III studies, which investigate a switch from one TKI to another, are ongoing. Pazopanib, sorafenib, axitinib, cediranib and sunitinib are all being investigated in this setting. Data with axitinib is likely to be the first of these studies to report. Axitinib is a potent VEGF-specific target TKI with few ‘off target’ effects. Results in the post interferon setting are impressive (PFS of 15.7 months (95% CI: 8.4–23.4)) and a phase II study that investigated axitinib post sorafenib are particularly striking (PFS and OS were 7.4 months (95% CI: 6.7–11.0) and 13.6 months (95% CI: 8.4–18.8), respectively) (Rixe *et al*, 2007; Rini *et al*, 2009). A randomised phase III second line study, comparing sorafenib and axitinib post VEGF-targeted therapy is now closed and results are awaited. The occurrence of hypertension with axitinib may predict clinical benefit, which is being investigated formally in clinical trials (Rixe *et al*, 2008).

## Other agents under investigation

Tivozinib is a potent VEGF TKI with impressive clinical data. In a randomised discontinuation study 88% of patients obtained a clinical benefit from the drug (Bhargava *et al*, 2009). A randomised phase III study comparing tivozinib and sorafenib in patients not previously exposed to targeted therapy will close in December 2010. Dovitinib is another promising TKI that targets FGF-2, as well as VEGF. It is being investigated in the third line setting in mTOR refractory disease.

## Combination therapy

A logical step in improving outcomes is to investigate these drugs in combination. However, combining sunitinib with other forms of targeted therapy has been difficult because of excessive toxicity (Feldman *et al*, 2009). This may not be the case with bevacizumab, which has been shown to be tolerable in combination with other agents such as everolimus. In a phase II study of bevacizumab and everolimus the median PFS for this combination was 9.1 months with response rates of 30% (Hainsworth *et al*, 2010). A recent randomised phase II study comparing sunitinib v.s. bevacizumab and interferon v.s bevacizumab and temsirolimus demonstrated significant toxicity for the latter combination without any efficacy advantage (Escudier *et al*, 2010). Perhaps, the most early awaited combination data is RECORD II that compares bevacizumab and everolimus *vs* bevacizumab and interferon. The potential disadvantages of combination therapy include additional toxicity, cost and the theoretical risk of multidrug resistance. Early results in this setting have been disappointing.

## Mechanisms of resistance to therapy

The mutation to the *VHL* gene and subsequent activation of HIF and VEGF are hall marks of clear cell renal cancer. The VEGF-targeted therapy is thought to work by blocking the angiogenic effects of this overactive pathway (Gossage and Eisen, 2010). Only a small minority of renal tumours initially progress through VEGF-targeted therapy, and the majority of patients initially obtain clinical benefit with these drugs (Motzer *et al*, 2009). However, resistance occurs, which is usually referred to as acquired resistance (Rini and Atkins, 2009). The onset of this acquired resistance is variable both in terms of time and clinical pattern, and our understanding of it at the molecular level is in its infancy. This is largely due to the lack of sequential tissue for comparative analysis.

Unlike in other malignancies, the acquisition of specific mutation to the target of the drug is not thought to be responsible for resistance in RCC (Valent, 2008; Rini *et al*, 2009). This is because in RCC the main target for sunitinib is the VEGF receptors on the vascular endothelium that are genetically stable.

There are currently two main theories to account for acquired resistance. First, it is speculated that ‘angiogenic escape’ occurs, in which with time the initial targeted therapy becomes ineffective at blocking the VEGF axis. This hypothesis is supported by the observation that switching from one VEGF-targeted therapy to another potentially more potent agent results in further responses (Rini *et al*, 2009). It is speculated that this may be due to upregulation of associated growth factors, such as HIF1-*α* (Rini *et al*, 2009). The second area of investigation is based around the emerging preclinical data that support the role of alternative pathways and growth factors in propagating tumour growth in acquired resistance. Specifically, FGF-2, tie2/Ang2, il-8 and the Src family have been implicated in this process (Oliner *et al*, 2004; Cenni *et al*, 2005; Bergers and Hanahan 2008). Clinical trials have been designed to target all these protein with dovotinib, AMG386 and saracatinib. Resistance also occurs universally with the mTOR inhibitors. The current drugs in mRCC only target TORC1 and it appears that inhibition of this in isolation leads to compensatory upregulation of PI3 kinase (O’Reilly *et al*, 2006). The development of combined TORC1 and 2 inhibitors could potentially overcome this and will be investigated in mRCC (Sarbassov *et al*, 2006).

## Clinical trials in the future

The two main goals over the next 5–10 years in mRCC are to firmly establish the most effective agents in each class and identify predictive biomarkers associated with clinical benefit to specific agents. To achieve these goals two different trial designs are required. Randomised phase III studies comparing newer agents with benchmark controls (currently sunitinib for the first line and everolimus for second line) are required to redefine standard therapy. The COMPARZ study, a non-inferiority study, which compares pazopanib and sunitinib, has just closed and is due to report in 2011/12. Results are eagerly awaited by further studies with tivozinib and axitinib *vs* a benchmark are required before there is clarity about the optimal first line VEGF TKI. The assumption that these agents are more active in the first-line setting without the appropriate studies is flawed, despite their impressive efficacies in other settings. Sorafenib should not be considered an adequate benchmark control in the first line setting (Escudier *et al*, 2009a, 2009b, 2009c).

There is also a need for smaller biomarker studies, which take sequential tissue, plasma and functional imaging during therapy. These studies are aimed at gaining a better understanding of how each drug works. The true goal for these studies is to identify predictive biomarkers for specific agents, which requires randomisation against other agents.

## Conclusions

Targeted therapy has changed the way metastatic renal cancer is treated and the OS for these patients is now greater than 2 years in prospective studies. Sequential therapy with VEGF-targeted therapy and mTOR inhibitors are currently the standard of care. Data on specific combinations are eagerly awaited. Correlative markers associated with clinical benefit remain elusive and are urgently required to develop treatment for this disease. This will help us to move away from the current ‘one size fits all’ approach and help develop truly individualised targeted therapy.

## Figures and Tables

**Table 1:** Randomised studies in the first line setting in metastatic clear cell renal cancer

**Reference**	**Histology**	**MSKCC risk groups**	**Study drug *vs* control arm**	**Progression-free survival (months)** a	**Overall survival (months)** a
Motzer *et al*, 2007	Clear cell	All	Sunitinib *vs* interferon	11 *vs* 5	26.4 *vs* 21.8
Hudes *et al*, 2007	All types	Poor	Temsirolimus *vs* interferon	5.5 *vs* 3.1	10.9 *vs* 7.3
Escudier *et al*, 2007a, 2007b	Clear cell	All	Bevacizumab + interferon *vs* interferon	10.2 *vs* 5.4	22.9 *vs* 20.6
Rini *et al*, 2008a, 2008b	Clear cell	All	Bevacizumab +interferon *vs* interferon	8.5 *vs* 5.1	18.3 *vs* 17.4
Sternberg *et al*, 2009	Clear cell	All	Pazopanib *vs* placebo	11.1 *vs* 2.8	NA

a The first figure is that of the study arm the second figure is for the control arm.
